# Promiscuous DNA cleavage by HpyAII endonuclease is modulated by the HNH catalytic residues

**DOI:** 10.1042/BSR20201633

**Published:** 2020-09-16

**Authors:** Sumith Kumar, Sushant Bangru, Ritesh Kumar, Desirazu N. Rao

**Affiliations:** 1 Department of Biochemistry, Indian Institute of Science, CV Raman Road, Bangalore, India; 2 Jawarharlal Nehru Center for Advanced Scientific Research, Jakkur, Bangalore-560064, India

**Keywords:** directed evolution, endonucleases, Helicobacter pylori, restriction-modification system, site-directed mutagenesis

## Abstract

*Helicobacter pylori* is a carcinogenic bacterium that is responsible for 5.5% of all human gastric cancers. *H. pylori* codes for an unusually large number of restriction–modification (R–M) systems and several of them are strain-specific and phase-variable. HpyAII is a novel Type IIs phase-variable restriction endonuclease present in 26695 strain of *H. pylori*. We show that HpyAII prefers two-site substrates over one-site substrates for maximal cleavage activity. HpyAII is less stringent in metal ion requirement and shows higher cleavage activity with Ni^2+^ over Mg^2+^. Mutational analysis of the putative residues of the HNH motif of HpyAII confirms that the protein has an active HNH site for the cleavage of DNA. However, mutation of the first Histidine residue of the HNH motif to Alanine does not abolish the enzymatic activity, but instead causes loss of fidelity compared with wildtype HpyAII. Previous studies have shown that mutation of the first Histidine residue of the HNH motif of all other known HNH motif motif-containing enzymes completely abolishes enzymatic activity. We found, in the case of HpyAII, mutation of an active site residue leads to the loss of endonuclease fidelity. The present study provides further insights into the evolution of restriction enzymes.

## Introduction

*Helicobacter pylori* is a Gram-negative, microaerophilic, spiral-shaped bacterium known to cause multiple gastric diseases such as peptic and duodenal ulcers, gastritis, and gastric cancer in humans. High rates of mutations and natural competence are factors responsible for the high level of allelic diversity in *H. pylori*. Recent methylome studies have revealed the abundance of multiple Restriction–Modification (R–M) systems and presence of strain-specific DNA methylation pattern in *H. pylori* strains [1,2].

R–M systems evolved as a defense mechanism against invading foreign DNA, however, studies have shown that components of R–M systems control virulence and gene expression in pathogenic bacteria [3–9]. R–M systems in *H. pylori* act as mobile genetic elements with a mechanism similar to classical transposon insertion where insertion with long target duplication results in mobility of R–M systems [10]. However, many R–M systems can become inactive due to random insertions, deletions and point mutations [11,12]. *H. pylori* 26695 strain bears two active Type IIs R–M systems, namely HpyAII and HpyAV [1]. However, HpyAII R–M system is subjected to a novel type of regulation by gene deletion and horizontal reconstitution [13], where a recombination event at 80-bp flanking repeat sequence can either delete or restore the R–M system in the genome. HpyAII R–M system is composed of two exocyclic DNA methyltransferases (MTases) (M1.HpyAII and M2.HpyAII), and a novel phase-variable type IIs HpyAII endonuclease. HpyAII endonuclease recognizes the sequence 5′-GAAGA-3′/3′-CTTCT-5′ and cleaves 8- and 7-bp downstream of the recognition sequence [14]. M1.HpyAII methylates the last adenine residue in the GAAGA sequence and M2.HpyAII methylates the first cytosine residue at N-4 position in the TCTTC sequence in the complimentary strand [1]. Previously, we reported that N^4^-DNA cytosine methylation by M2.HpyAII plays an epigenetic role in *H. pylori* and regulates virulence and gene expression [8].

Since HpyAII is a type IIs endonuclease, it cleaves the DNA outside the recognition site thus leaving the recognition site intact. Upon cleavage, type IIs endonucleases create DNA fragments with overhangs unrelated to the target sequence, thus making them useful tool in the assembly of DNA from small DNA fragments [15]. Bioinformatics analyses suggested that HpyAII endonuclease active site does not belong to the more common PD…D/EXK superfamily but harbors a ββα-Me-finger fold, characteristic of the HNH superfamily of nucleases. Interestingly, the isoschizomer of HpyAII, MboII which recognizes and cleaves the DNA at the identical site, contains a PD…D/EXK motif [16] indicating that the two enzymes might have different kinetic mechanisms utilizing different catalytic residues for DNA cleavage.

Till date, very few type IIs endonucleases are known for which complete reaction pathway has been studied [17]. Considering the diversity of type IIs endonuclease regarding cofactors requirements and substrate preferences, it is unlikely that different type IIs endonucleases will act in the same way. In the present study, we studied substrate preference, cofactor requirement, identification and analysis of the active-site residues of the novel phase-variable HpyAII endonuclease.

## Experimental procedures

### Bacterial strains, plasmid vectors, chemicals and enzymes

*Escherichia coli* DH5α [F′ end A1 hsd R17 (rk- mk -) glnV44 thi1 recA1 gyrA (NalR) relA1 Δ (lacIZYA – argF) U169 deoR (Φ80dlac Δ (lacZ) M15}] strain was used as a host for preparing pUC19, pET28a, pMS1, pMS10A, and recombinant DNA constructs. *E. coli* Rosetta (DE3) pLysS [(F- ompT hsdSB(RB – mB) gal dcm λ(DE3 [lacI lacUV5-T7 gene 1 ind1 sam7 nin5]) pLysSRARE (CamR)] strain was used for overexpression and purification of HpyAII endonuclease and its mutant proteins. All strains were cultured in lysogeny broth (LB) at 37°C or mentioned temperatures. All reagents used were of analytical or ultrapure grade. Restriction enzymes, DNA polymerases, T4 DNA ligase and T4 polynucleotide kinase were used according to manufacturers’ protocols.

### Cloning and construction of HpyAII restriction endonuclease

*hpyAII* ORF (1272 bp) was amplified from genomic DNA of *H. pylori* strain 26695 by polymerase chain reaction (PCR) using Q5 polymerase with primers 1 and 2 (Table 1). The primers were designed with the help of the annotated complete genome sequence of *H. pylori* 26695, considering the putative gene sequence of *hpyAII* obtained from The Institute for Genomic Research (TIGR). The amplified PCR product was purified from agarose gel and digested with BamHI and XhoI. The digested PCR product was purified using PCR purification kit and ligated with BamHI-XhoI digested pET28a vector. The ligated recombinant pET28a plasmid has the *hpyAII* ORF in the Multiple Cloning Site (MCS) region along with N-terminal hexa-histidine (His_6_) tag under isopropyl-1-thio-β-d-galactopyranoside (IPTG)-inducible promoter. The recombinant plasmid was transformed into *E. coli* DH5α cells and selected with kanamycin. The clones were confirmed by performing restriction digestion with BamHI and XhoI and further confirmed by DNA sequencing.

**Table 1:** Oligonucleotides used in the study

Primer	Primer name	Nucleotide sequence 5′–3′
1	hpyAIIF	CATATGATGCCAAAATTAGAAAAAATTTTGC
2.	hpyAIIR	GGATCC TCAGCCGTTTTTAGAATGCAAT
3.	60F	CCTTGTTTTGAAGAATGTTTTGCTTTGAAAATTTGAATATTCGCGGATCCAATGTTTTGC
4.	60R	GGAACAAAACTTCTTACAAAACGAAACTTTTAAACTTATAAGCGCCTAGGTTACAAAACG
5.	Two-site F	TGTTTTGTTTGTTTTGAAGATGCCTCATGTAGGGAGCAAAACATAAGGCATCTTCAAAACAAACGAAAT
6.	Two-site R	ACAAACAAAACTTCTACGGAGTACATCCCTCGTTTTGTATTCCGTAGAAGTTTTGTTTGC
7.	Star1 F	GCAAAACATTGGATCCGCGAATATTCAAATTTTCAAAGCAAAACATTCTCCAAAACAAGG
8.	Star1 R	CCTTGTTTTGGAGAATGTTTTGCTTTGAAAATTTGAATATTCGCGGATCCAATGTTTTGC
9.	Star2 F	GCAAAACATTGGATCCGCGAATATTCAAATTTTCAAAGCAAAACATTCCTCAAAACAAGG
10.	Star2 R	CCTTGTTTTGATGAATGTTTTGCTTTGAAAATTTGAATATTCGCGGATCCAATGTTTTGC
11.	Star3 F	GCAAAACATTGGATCCGCGAATATTCAAATTTTCAAAGCAAAACATTTTTCAAAACAAGG
12.	Star3 R	CCTTGTTTTGAAAAATGTTTTGCTTTGAAAATTTGAATATTCGCGGATCCAATGTTTTGC
13.	H328A F	CTTTGAATTGGCCCACATTGTGCCTTTATGCTTG*GCC*CGCTCTATAG
14.	H329A F	GAAAAGGGCTTTGAATTGCACGCCATTGTGCCTTTATGCTTGG
15.	H329A R	CCAAGCATAAAGGCACAATGGCGTGCAATTCAAAGCCCTTTTC
16.	H359A F	CTATATTGACGCTTTTAACGCTGCGAAAATATCTCAAACGC
17.	H359A R	GCGTTTGAGATATTTTCGCAGCGTTAAAAGCGTCAATATAG
18.	H359Q F	CTATATTGACGCTTTTAACCAAGCGAAAATATCTCAAACGC
19.	H359Q R	GCGTTTGAGATATTTTCGCTTGGTTAAAAGCGTCAATATAG

### Overexpression and purification of HpyAII endonuclease

HpyAII endonuclease was overexpressed by transforming the recombinant plasmid in *E. coli* Rosetta (DE3) pLysS strain. Cells picked from a single colony after transformation were grown overnight in LB broth containing 45 µg/ml of kanamycin and 35 µg/ml of chloramphenicol at 37°C. One percent of the primary inoculum was then used for the inoculation of LB broth containing 20 mM glucose, 45 µg/ml kanamycin and 35 µg/ml chloramphenicol. Cells were grown at 37°C for O.D._600 nm_ 0.8–1.0 and induced with 1 mM IPTG. After 30 min of induction, 100 µg/ml rifampicin was added to inhibit the host *E. coli* RNA polymerase. Induced culture was grown for another 2 h at 37°C. Cells were harvested by centrifugation at 5000×***g*** for 10 min at 4°C. The bacterial pellet was resuspended in buffer A (10 mM Tris-Cl pH 7.4, 100 mM NaCl, 2 mM β-mercaptoethanol, protease inhibitor cocktail (1×) and 10% glycerol) and lysed by sonication. The cell lysate was cleared by centrifugation at 14000 rpm for 30 min 4°C. Ni^2+^-NTA agarose (3 ml bed volume) equilibrated in the lysis buffer was added to the supernatant, and the proteins were allowed to bind for 3 h on ice with intermittent mixing. The protein-bound matrix was packed into a column and washed with at least 10 column volumes of wash buffer (buffer A containing 50 mM imidazole). The proteins were eluted using a gradient of 50–400 mM imidazole and checked on SDS-10% polyacrylamide gel. The eluted protein was dialyzed extensively against the buffer B (10 mM Tris-Cl, pH 7.4, 5 mM β-mercaptoethanol, 100 mM NaCl, 10% glycerol). Dialyzed sample was loaded on pre-equilibrated HiTrap heparin column (GE Healthcare), and binding was performed at 1 ml.min^−1^. The column was washed with 50 column volumes of buffer B and protein was eluted using buffer B with an NaCl gradient of 0.1–1.0 M. Eluted samples were pooled and desalted using HiTrap desalting column (GE Healthcare) equilibrated with buffer B. Desalted samples were further purified using HiTrap SP HP column (GE Healthcare) in buffer B. The column was washed with 50 volumes of buffer B. Elution of HpyAII was performed using NaCl gradient (0.1–1 M). The purity of the purified protein was analyzed on the SDS-10% polyacrylamide gel. Purified pooled fractions were first dialyzed in buffer B, followed by dialysis in storage buffer containing 50% glycerol. Dialyzed samples were kept in −20°C for further analysis. Protein concentration was determined by performing Bradford’s assay using bovine serum albumin as standard.

### Mass spectroscopy and peptide mass fingerprinting

Gel trypsin digestion of HpyAII endonuclease was performed as described [18]. MALDI-MS data were acquired on Ultraflex TOF/TOF spectrometer (Bruker Daltonics, Billericia, MA, U.S.A. and Bremen, Germany), equipped with a 50-Hz pulsed nitrogen laser (1^1^/_4_337 nm), operated in positive ion reflection mode using a 90-ns time delay and a 25-kV accelerating voltage. The samples were prepared by mixing equal amount of peptide with matrices dihydroxybenzoic acid/α-cyano-4 hydroxycinnamic acid saturated in 0.1% trifluoroacetic acid and acetonitrile (1:1 v/v). Masses below 500 m/z were not considered as a result of interference from the matrix.

### DNA substrates preparation

HpyAII DNA oligonucleotide substrates carrying a different number of 5′ GAAGA 3′ sites (primer 3-12, Table 1) were prepared by annealing various combinations of chemically synthesized oligonucleotides (Sigma Genosys) (Table 1). Individual single-stranded oligonucleotides were radiolabeled at the 5′-end with [γ-^32^P] ATP using T4 polynucleotide kinase (NEB) and purified using QIAGEN nucleotide removal kit. The different double-stranded DNA substrates were prepared by mixing 1.5-fold molar excess of unlabeled complementary oligonucleotides with the radiolabeled oligonucleotides (Table 1). The mixture was boiled for 10 min at 95°C in 1X saline-sodium citrate (SSC) buffer (15 mM trisodium citrate, pH 7.0 and 150 mM sodium chloride). Samples were allowed to cool to room temperature after annealing. Annealed DNA substrates were electrophoresed on 10% native polyacrylamide gel in 1× TBE buffer. Autoradiography was performed to identify the region containing the double-stranded DNA substrates, gel pieces were excised out to elute DNA substrate in 1× TE buffer (10 mM Tris-HCl and 1 mM EDTA).

Derivatives of pUC18 plasmid with one GAAGA site (pMS1) and two GAAGA sites (pMS10A) were obtained as a kind gift from Prof. Bernard A. Connolly, Institute for Cell and Molecular Biosciences, Newcastle University, Newcastle upon Tyne NE2 4HH, (UK)(Supplementary Figure S1) [16]. Both the plasmids were grown in *E. coli* DH5α strain and purified from 100-ml bacterial culture using QIAGEN plasmid purification kit. The concentration of the purified plasmids was estimated by measuring absorbance at 260 nm.

### DNA cleavage assays

A unit of HpyAII endonuclease was determined by performing cleavage of 1 µg λ DNA (unmethylated). Different concentrations (10–300 ng) of HpyAII endonuclease were used for the cleavage assay. One unit was defined as the amount of HpyAII required to completely cleave 1 µg λDNA in a 50-µl reaction volume containing 10 mM MgCl_2_ for 1 h at 37°C. As a control, 1 unit of MboII was used to generate the complete digestion profile of λDNA.

Cleavage of pMS1 and pMS10A was carried out in 150 µl reaction volume in cleavage buffer (10 mM Tris-Cl, pH 7.0, 1 mM MgCl_2_ and 1 mM DTT) at 37°C. DNA cleavage was performed under single turnover conditions ([E] > [S]). The concentration of the supercoiled plasmid DNA was 10 nM and the reaction was started by the addition of HpyAII to a final concentration 200 nM. After indicated time interval, 10-µl aliquots were withdrawn and mixed with reaction stopping solution (10 mM Tris-Cl, pH 8.0, 20 mM EDTA, 30% glycerol and 100 µg.ml^−1^ Orange G). The samples were analyzed by electrophoresis on 1% agarose gel containing 0.5 µg.ml^−1^ Ethidium Bromide, in 1× TAE buffer. DNA bands were visualized in AlphaImager MultiImage™ Light Cabinet (Alpha Innotech Corporation). The gels were visualized by phosphorimaging software and quantified using Multi Gauge version 2.3 (Fujifilm, Tokyo, Japan).

Cleavage of oligonucleotide substrates was carried out in 130 µl reaction volume in cleavage buffer (10 mM Tris-Cl, pH 7.0, 1 mM MgCl_2_ and 1 mM DTT) at 37°C. The final concentration of labeled DNA substrate was 1 nM and the reaction was started by the addition of enzyme to a final concentration of 200 nM. Zero-time point was taken before the addition of the enzyme and 10 µl was removed. At indicated time points, reaction progress was monitored by removing 10-µl aliquots and mixing them with reaction stop buffer (98% formamide, 0.1% Bromophenol Blue, 0.1% xylene cyanol, 0.1% Orange G and 20 mM EDTA). Samples were analyzed by denaturing gel electrophoresis (15%, 19:1 acrylamide/bis-acrylamide with 7 M urea) in 1× TBE buffer (89 mM Tris-borate, pH 8.3 and 20 mM EDTA). Gels were run at 300 V for 120 min and after electrophoresis, were transferred on to Whatman 3 mm paper and dried under vacuum at 80°C for 45 min. The gels were visualized by phosphorimaging and quantified using Multi Gauge version 2.3 (Fujifilm, Tokyo, Japan).

### Construction and purification of HpyAII endonuclease mutants using site-directed mutagenesis

Point-mutants of HpyAII endonuclease were constructed using QuickChange II Site-Directed Mutagenesis Kit (Agilent Technologies). Primers were designed and PCR were performed according to the manufacturer’s protocol. HpyAII H328A, H329A, H359A and H359Q mutants were constructed using primers 13-19 (Table 1). All the mutants were transformed in *E. coli* Rosetta (DE3) pLysS strain for overexpression and purification. Purification of the mutant proteins were performed as described for the HpyAII endonuclease.

### Circular dichroism measurements

Circular dichroism (CD) measurements were measured on a Jasco J810 polarimeter between 200 and 300 nm in a quartz cuvette of 1 mm path length. The spectra were recorded from 200 to 300 nm with 20 mdeg sensitivity at a scanning speed of 100 nm per minute. All experiments were done with 2 µM protein samples in 10 mM Tris-HCl buffer (pH 7.4) at 20°C. The CD spectral data consist of three scans after correction for the buffer baseline and were reported as mean residue ellipticity [θ].

Following equation was used to obtain mean residual ellipticity [θ MRE] values from ellipticity values.

$${\left[\theta \right]}_{\text{MRE}}=\frac{\theta \times 100\times \text{Mr}}{\text{C}\times \text{L}\times \text{NA}}$$

where, [θ]_MRE_ is mean residual ellipticity (degrees), θ is observed ellipticity, Mr is molecular weight of the proteins in Da, L is path length in cm and NA is number of amino acids.

### Statistical analysis of data

Experiments were performed at least in duplicates and repeated twice. All the analyses were performed using GraphPad Prism software version 6.0 (GraphPad software Inc., La Jolla, CA, U.S.A.).

## Results

### Cloning, overexpression and purification of HpyAII endonuclease

*E. coli* Rosetta (DE3) pLysS cells were transformed with recombinant pET28a plasmid containing *hpyAII* ORF. To avoid the toxicity due to the overexpression of HpyAII restriction endonuclease in the absence of cognate methyltransferases, a modified protocol was used [14]. Cells were induced at high cell density (O.D._600_ 0.8–1.0) with 1 mM IPTG. After 30 min of induction, rifampicin (100 µg/ml) was added to the cell cultures. Cells were further grown for 2 h at 37°C after addition of rifampicin. The addition of rifampicin reduces the expression of *E. coli* proteins by selectively inhibiting the *E. coli* RNA polymerase, whereas T7 RNA polymerase will be refractory to rifampicin action [19]. A polypeptide of expected molecular weight of His_6_-tag HpyAII (53 kDa) was expressed after induction with IPTG (Supplementary Figure S2A). However, a notable reduction in the *E. coli* proteins was observed in the induced samples. This reduction was seen due to the selective inhibition of host RNA polymerase by rifampicin. The purity of the purified protein was evaluated by performing silver staining analysis along with peptide mass fingerprinting (Supplementary Figures S2B and S3).

### Multiple sequence alignment of HpyAII endonuclease

**Figure 1:**
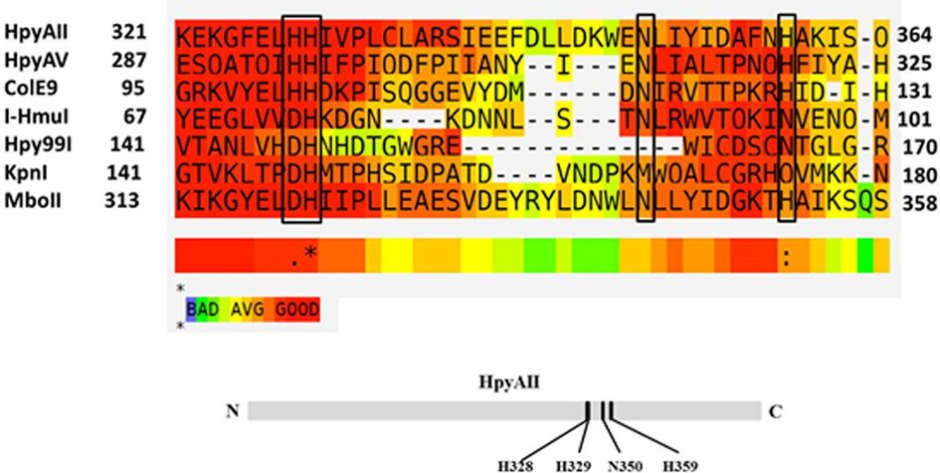
Multiple sequence alignment of HpyAII endonuclease with HNH motif containing enzymes Shading represents the quality of the alignment. Alignment was made using T-COFFEE alignment on EMBnet server. Putative HNH motif residues of HpyAII endonuclease were identified as H328, H329, N350 and H359.

To identify the active-site residue(s) of HpyAII endonuclease, multiple sequence alignment was performed with restriction endonucleases having different catalytic domains. It was observed that HpyAII aligned well with HNH motif containing enzymes (Figure 1). The conserved residues of the putative HNH motif upon sequence alignment were identified as H328, H329, N350 and H359. HpyAII was found to have classical HNH motif with conserved His-His-X_n_-Asn-His residues (Figure 1).

### Cleavage of one-site and two-site plasmid DNA substrate

**Figure 2:**
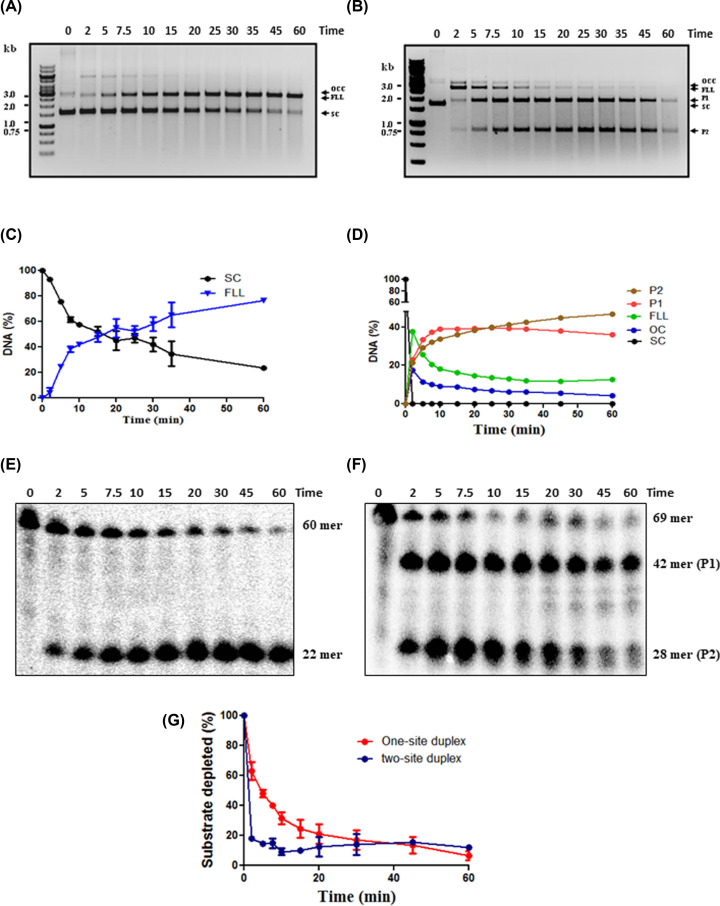
Cleavage of one-site and two-site supercoiled plasmid and oligonucleotide duplex DNA substrate by HpyAII endonuclease Cleavage of one-site supercoiled plasmid (pMS1) (**A**) and two-sites plasmid (pMS10A) (**B**) by HpyAII endonuclease in cleavage buffer supplemented with 1 mM MgCl_2_. Cleavage was done under single turnover conditions (200 nM enzyme and 10 nM plasmid DNA) at 37°C. Aliquots were removed at time intervals mentioned, mixed with reaction stop mixture and cleavage products were analyzed on 1% agarose gel. (**C**) Quantitation of cleavage of one-site plasmid; SC: supercoiled plasmid DNA, FLL. (**D**) Quantitation of cleavage of two-sites plasmid. Hydrolysis of the [^32^γ-P] labeled 60-bp oligo-duplex (containing one site) P1: product 1, P2: product 2, FLL, OC: open circular plasmid and SC: supercoiled plasmid DNA (**E**) and 69-bp two-site oligo duplex (**F**) by HpyAII was carried out under single turnover reaction conditions (200 nM enzyme and 1 nM DNA) at 37°C in the cleavage buffer supplemented with 1 mM MgCl_2_. Aliquots were removed at time intervals mentioned, mixed with reaction stop mixture and cleavage products were analyzed by denaturing gel electrophoresis (12% polyacrylamide gel containing 8 M urea). Under denaturing conditions, double-stranded DNA product will be separated into single strands and only radiolabeled single strand DNA (22-mer) will be visible on the gel. The intensity of each band (P1 and P2) was estimated as mentioned in the ‘Experimental procedures’ section. (**G**) Quantitation of one-site and two-site DNA substrate hydrolysis from densitometric analysis of denaturing PAGE gel. SC, supercoiled plasmid.

Type IIs restriction enzymes recognize an asymmetric DNA sequence (4–7 bp) and cleave DNA at both the strands downstream of the recognition sequence within 20 bp [20]. The cleavage activity of HpyAII endonuclease was monitored using one- and two-site supercoiled plasmid DNA (Figure 2). It was observed that HpyAII was unable to cleave one- or two-site substrate when the enzyme concentration was lower compared with the substrate concentration (data not shown). However, the enzyme was active on both one- and two-site supercoiled plasmid substrate in a single turn-over reaction conditions ([E] > [S]). The cleavage of one-site supercoiled plasmid (pMS1) occurred at a slower rate compared with the two-site supercoiled plasmid (pMS10A) (Figure 2). Incubation of excess of HpyAII endonuclease (200 nM) with 10 nM of pMS1 resulted in the cleavage of approximately 80% of the plasmid DNA in 60 min (Figure 2A,C). Cleavage of pMS1 to the full-length linear fragment (FLL) was accompanied by the negligible formation of open-circular plasmid (Figure 2A).

The halfway point of the reaction, at which concentration of substrate and product are equal, was found to be 15 min for pMS1 plasmid (Figure 2C). The cleavage of pMS10A under similar reaction conditions was much more rapid, and within 2 min of incubation, complete conversion of supercoiled plasmid DNA into FLL was observed. The complete cleavage of pMS10A to fragments P1 (1842 bp) and P2 (844 bp) was accompanied by the transient appearance of open-circular plasmid DNA (OC) (resulting from the cleavage of a single strand) (Figure 2B,D). The flanking sequence of the two sites in pMS10A is identical with preclude any potential sequence bias for cleavage [16]. It was observed that HpyAII was much more active on pMS10A compared with pMS1 (Figure 2C,D) indicating that HpyAII endonuclease requires two-sites in the same plasmid for maximal activity. Due to the rapid cleavage of pMS10A, an accurate estimation of the half-life of the reaction was not possible.

### Cleavage of one-site and two-site oligonucleotide substrate

To confirm the low cleavage activity of HpyAII endonuclease on one-site plasmid substrate, oligonucleotide substrates carrying one or two recognition sites were used. To monitor the cleavage, a one-site 60-bp oligonucleotide duplex (1 nM) was used as substrate. The bottom strand was radiolabeled with [^32^P]-ATP at 5′ end. Complete cleavage of this substrate will produce two fragments of size 22 (P1) and 38 bp (P2) (Supplementary Figure S1) but only the 22-mer single strand will be visible on denaturing PAGE upon autoradiography. Incubation of excess amount of HpyAII (200 nM) with 1 nM of radiolabeled one-site substrate resulted modest DNA cleavage. Uncleaved DNA substrate was visible even after 60 min (Figure 2E). However, two-site substrate under similar conditions was cleaved much more rapidly (Figure 2F). The halfway point of the reaction was defined as a time point where percentage proportion of substrate and products was found to be equal. For one side, substrate half way point was found to be 5 min, whereas it was 30 s with two-site substrate (Figure 2F,G). This confirms the preference of HpyAII for two-site substrate for maximal activity. HpyAII cleaves both the sites of two-site oligonucleotide substrate simultaneously, since both P1 (42 bp) and P2 (28 bp) final products were generated at the same time (Figure 2E). At higher incubation time further degradation of P2 fragment was seen. This could be attributed to the star-activity of HpyAII due to extended incubation time.

#### Cofactor requirement of HpyAII endonuclease

**Figure 3:**
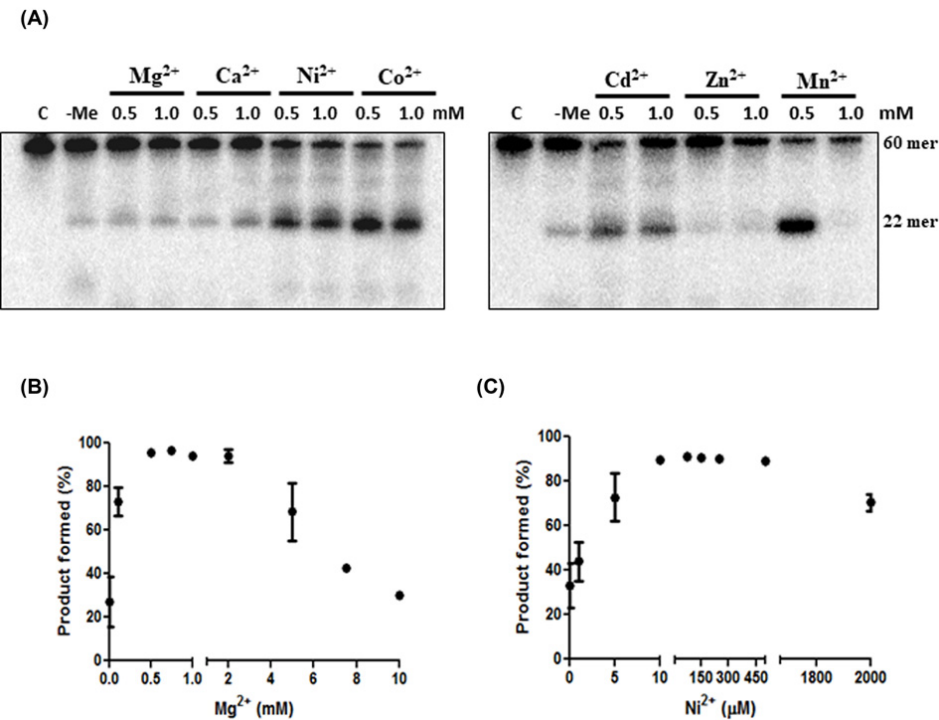
Effect of divalent metal ions on the cleavage activity of HpyAII endonuclease (**A**) Effect of divalent metal ions on HpyAII endonuclease activity was assessed by performing the cleavage of 60 bp one-site oligonucleotide duplex DNA at the indicated concentration of metal ions. [^32^γ-P] radiolabeled DNA (1 nM) was incubated with 40 nM HpyAII endonuclease in the cleavage buffer with various divalent metal ions. Cleavage of 60 bp DNA substrate resulted in 22-bp radiolabeled fragment. Reaction was performed at 37°C for 5 min and stopped by the addition of reaction stop mixture containing 20 mM EDTA. Effect of divalent metal ions on HpyAII endonuclease activity was assessed by performing the titration of experiment. (**B**) Mg^2+^ and (**C**) Ni^2+^ was varied from 0.1 µM to 2 mM in the reaction mixture. Cleavage of 60-bp one-site oligonucleotide duplex DNA was monitored at the indicated concentration of metal ions. [^32^γ-P] radiolabeled DNA (1 nM) was incubated with 40 nM HpyAII endonuclease in the cleavage buffer with various divalent metal ions. Reaction was performed at 37°C for 5 min and stopped by the addition of reaction stop mixture containing 20 mM EDTA. To monitor DNA cleavage, samples were analyzed on denaturing gel electrophoresis (12% polyacrylamide gel containing 8 M urea) and release of 22-bp cleavage product was monitored as radiolabeled single-stranded 22-mer product. Densitometric analysis of the denatured PAGE was done to obtain the relationship between metal ions concentration and product formed.

HpyAII exhibited low endonuclease activity on one-site oligonucleotide duplex substrate in the standard assay conditions containing Mg^2+^ ions. To investigate the low activity of HpyAII, cleavage of one-site substrate in the presence of different metal ions was assessed. Radiolabeled one-site oligonucleotide duplex substrate was incubated with different divalent metal ions (0.5 and 1.0 mM) at a constant HpyAII concentration. It was observed that HpyAII exhibited enhanced DNA cleavage in presence of Ni^2+^, Co^2+^, Cd^2+^, Mn^2+^ ions compared with Mg^2+^ and Ca^2+^ ions (Figure 3A). However, it was observed that Mn^2+^ had an inhibitory effect at 1.0 mM and no detectable activity was seen in presence of Zn^2+^ ion. HpyAII prefers transition metal ions over alkaline earth metals for maximal activity. It was demonstrated earlier that HpyAV, which is another type IIS restriction endonuclease, exhibits the same preference [21]. To further determine the preference of Ni^2+^ over Mg^2+^ ions for cleavage, titration experiment using one-site oligonucleotide duplex was performed. It was observed that approximately 90% of the substrate was cleaved in presence of 10 µM Ni^2+^ ions in 5 min (Figure 3B), whereas, a similar level of DNA cleavage was observed with 750 µM of Mg^2+^ ions (Figure 3C). However, at higher concentrations of Ni^2+^ (≥1.0 mM) (Figure 3B) and Mg^2+^ (≥ 2.0 mM) (Figure 3C) inhibition of DNA cleavage was observed. Results from these experiments suggest a substantial preference of Ni^2+^ ions over Mg^2+^ ions by HpyAII. A residual activity of HpyAII was seen in the absence of divalent metal ions (Figure 3). The reason for this residual cleavage activity could be bound metal ion or inherent ability of HpyAII to cleave DNA in the absence of metal ions. To further address this question, cleavage of radiolabeled one-site substrate was carried out in the absence of metal ions and increasing concentration of chelating agent EDTA. If the residual cleavage activity of HpyAII is independent of divalent metal ions then addition of EDTA should not affect the catalysis. However, if the cleavage is due to the presence of bound metal ion then DNA cleavage will be inhibited upon increase in EDTA concentration. Upon incubating the enzyme with radiolabeled DNA substrate it was observed that the residual HpyAII cleavage activity was sensitive to EDTA concentration and the cleavage was abolished at 2.0 mM EDTA concentration (Supplementary Figure S4A). Inhibition of the enzymatic activity was seen at low concentration of EDTA (0.1 mM) (Supplementary Figure S4B). However, the addition of metal ion (1.0 mM Mg^2+^) in the reaction mixture enhances the cleavage activity of HpyAII endonuclease on one-site substrate (Supplementary Figure S4A).

#### Analysis of HNH motif of HpyAII endonuclease

The alignment revealed that the amino acids H328, H329, N350 and H359 could constitute the putative HNH motif. To confirm the presence of HNH motif and verify the role of corresponding HNH residues of HpyAII endonuclease, site-directed mutagenesis (SDM) was performed. HpyAII H328A, H329A, H359Q and H359A mutant constructs were generated by SDM on the corresponding nucleotide sequence in the wildtype gene. The mutations were confirmed by sequencing. All mutants were overexpressed and purified by the same protocol that was used for the wildtype protein.

In the present study, the putative general acid residue (His^328^) was mutated to alanine. The H328A HpyAII was found to be soluble and purified to near homogeneity (Supplementary Figure S5A). CD spectra were collected for HpyAII and H328A HpyAII proteins to check the effect of H328A mutation on the secondary structure (Supplementary Figure S5B). There is no significant difference between the far CD spectra of HpyAII wildtype and H328A mutant suggesting that the mutation does not induce any gross structural changes in the protein.

**Figure 4:**
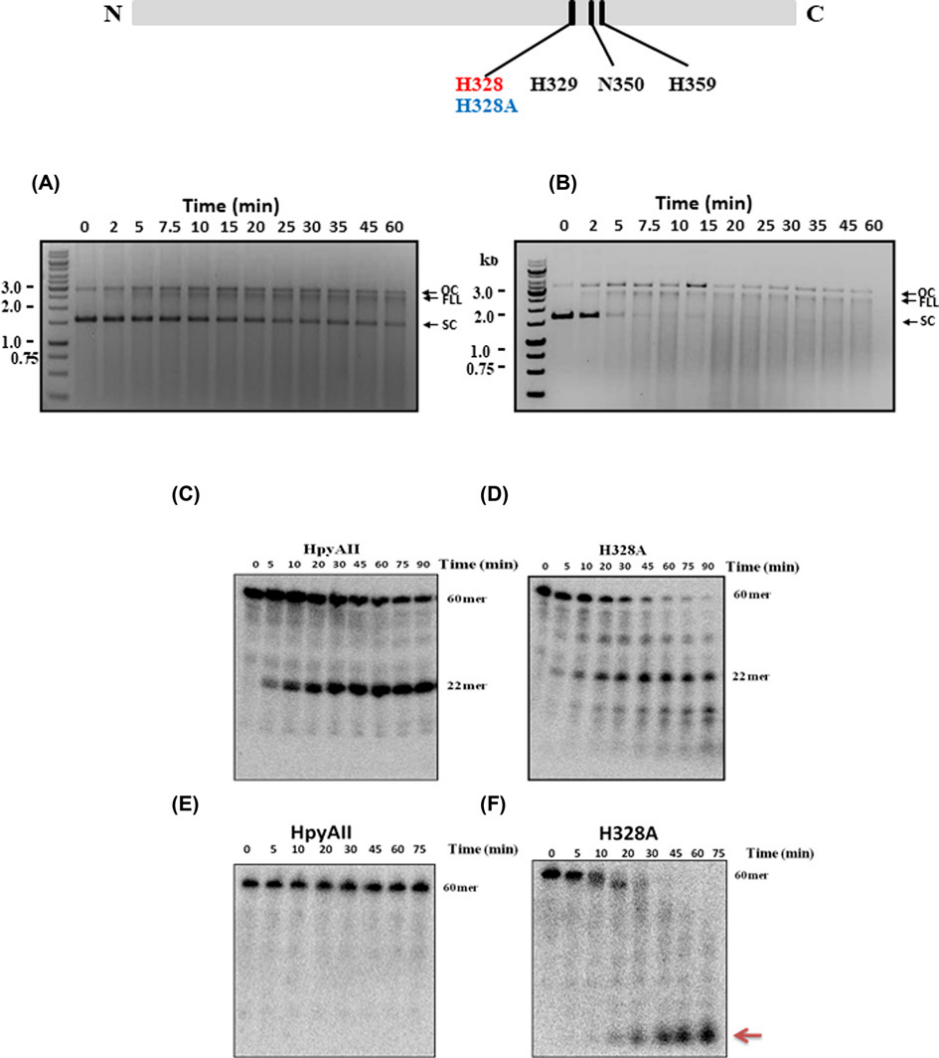
H328 residue is involved in fidelity of HpyAII endonuclease Cleavage of one-site (pMS1) (**A**) and two-site (pMS10A) (**B**) plasmid DNA substrate by H328A mutant enzyme. Cleavage was done under single turnover conditions (200 nM H328A mutant and 10 nM plasmid DNA) at 37°C. To monitor DNA cleavage aliquots were removed at the mentioned time intervals mixed with reaction stop mixture containing 20 mM EDTA. Cleavage products were analyzed on 1% agarose gel. Cleavage activity of HpyAII and H328A mutant were assessed on canonical (GAAGA) substrate (**C,D**) containing one-site and non-canonical (GGAGA) 60 bp duplex DNA (**E,F**). Substrates were radiolabeled with [^32^γ-P] and hydrolysis of 60 bp oligo-duplex was carried out under single turnover reaction condition. A total of 200 nM enzyme (HpyAII or H328A mutant) was incubated with 1 nM labeled DNA substrate and aliquots were removed at the time intervals mentioned. Reaction was stopped by the addition of reaction stop mixture containing 20 mM EDTA. Cleavage products were separated by performing denaturing gel electrophoresis (12% polyacrylamide gel containing 8 M urea). (**G**) Quantitation of canonical and non-canonical substrate cleavage by HpyAII and H328A mutant.

To understand the role of H328 residue, H328A mutant was assayed on one-site and two-site supercoiled plasmid DNA. It was observed that the mutation of Histidine at 328 position to Alanine resulted in active enzyme which can cleave both one-site (pMS1) and two-site (pMS10A) substrates in presence of Mg^2+^ ions. A similar cleavage profile like wildtype was seen with H328A mutant enzyme with pMS1. However, H328A mutation lead to increased accumulation of supercoiled substrate along and accumulation of open-circular plasmid (OC) (Figures 2A and 4A). In contrast when pMS10A was used as a substrate, H328A mutant showed no distinct cleavage pattern and non-specific cleavage was seen after 5 min of incubation time (Figure 4B). H328A mutation made the enzyme non-specific or promiscuous on the two-site plasmid DNA substrate. This result was in contrast with the previous observations where mutation of general acid residue of HNH motif always resulted in inactivation of enzymatic activity. H328A mutant was able to cleave pMS10A faster compared with the pMS1 supercoiled plasmid (Figure 4A,B) indicating the preference of H328A mutant enzyme for two-site plasmid substrate for maximal activity similar to wildtype HpyAII.

Both pMS1 and pMS10A were cleaved non-specifically by H328A mutant. However, it is difficult to determine whether the plasmid DNA is cleaved at the star-sites or at non-specific sequences. To address this question cleavage of radiolabeled one-site and star-site containing oligonucleotides was carried out in the presence of Mg^2+^ ions. It was observed that the H328A mutant can cleave one-site oligonucleotide substrate non-specifically unlike wildtype HpyAII (Figure 4C,D). The wildtype HpyAII cleaved the 60-bp DNA substrate to two fragments of which only 22-bp radiolabeled fragment was visible on denaturing PAGE. However, H328A mutant cleaved the 60-bp substrate DNA at the GAAGA site generating 22-bp fragment but also displayed promiscuous cleavage leading to smaller fragments (Figure 4C,D).

**Figure 5:**
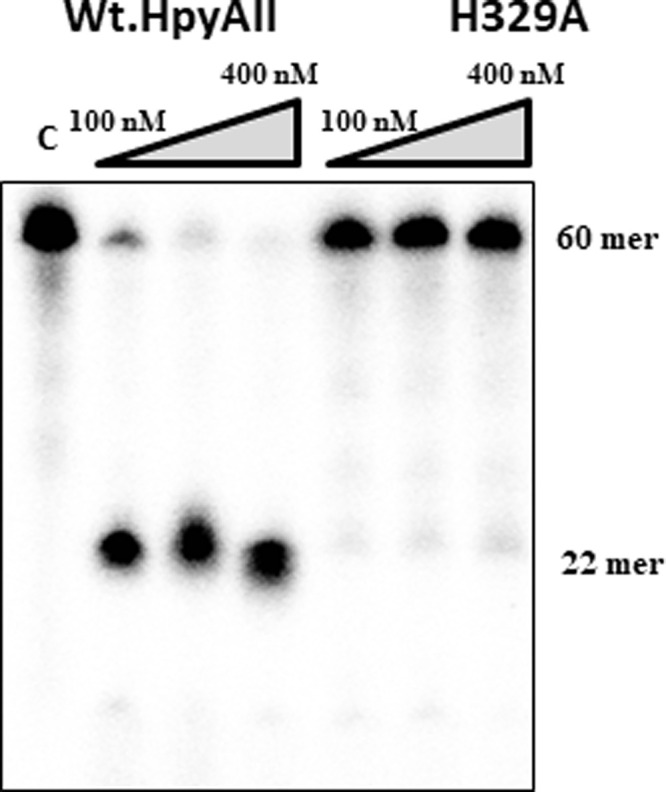
H329A mutation makes HpyAII endonuclease inactive Radiolabeled one-site oligonucleotide duplex (60 bp) DNA substrate was used to access the cleavage properties of HpyAII wildtype and H329A mutant. Cleavage assay was done by incubating indicated concentrations of HpyAII, and H329A enzymes with 1 nM [^32-γ^P] labeled DNA substrate in the cleavage buffer containing 1 mM MgCl_2_. Cleavage of DNA substrate was monitored by denaturing gel electrophoresis as mentioned earlier.

H328A mutant endonuclease activity was further checked on a 60-bp substrate in which the canonical GAAGA site was mutated to GGAGA (non-canonical sequence substrate). Upon incubation with non-canonical recognition sequence substrate it was observed that H328A mutant cleaved the DNA non-specifically and smaller fragments were generated, whereas the wildtype HpyAII was unable to cleave the non-canonical sequence substrate under similar conditions (Figure 4E,F). The single point mutation in HpyAII generated a non-specific mutant which can cleave at the non-canonical recognition sequence. It was also observed that the H328A mutant cleaved the non-canonical sequence substrate faster compared with the canonical one-site substrate (Figure 4F). These results suggest that Histidine at 328 position of HpyAII, which is a part of HNH motif, regulates the fidelity of the HpyAII. The presence of His^328^ is essential for the fidelity of the enzyme and prevents cleavage at non-canonical HpyAII DNA recognition sites. However, mutation of the H329 to Alanine resulted into inactive enzyme with no detectable cleavage seen with 60-bp DNA substrate containing one cleavage site (Figure 5). Inactivation of enzymatic activity by H329A mutation confirms that HpyAII utilizes HNH motif for DNA cleavage and His at 329 position is essential for the enzymatic activity.

**Figure 6:**
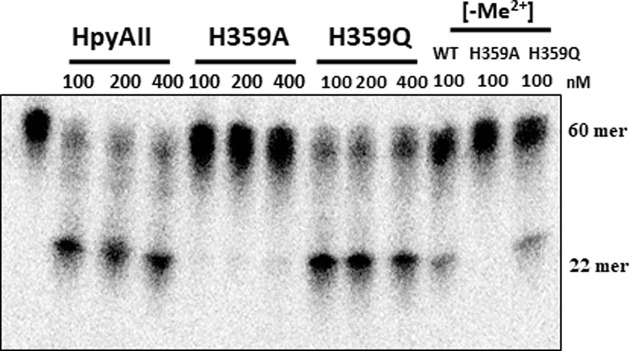
Cleavage activities of H359A and H359Q mutants of HpyAII endonuclease Radiolabeled one-site oligonucleotide duplex (60 bp) DNA substrate was used to access the cleavage properties of H359A and H359Q mutants. Cleavage assay was done by incubating indicated concentrations of HpyAII, H359A and H359Q enzymes with 1 nM [^32^-γP] labeled DNA substrate in the cleavage buffer containing 1 mM MgCl_2_. [-Me^2+^] indicate the divalent metal ion control reaction where divalent metal ion is absent from the reaction conditions. Cleavage of DNA substrate was monitored by denaturing gel electrophoresis as mentioned earlier.

HpyAII displayed some residual cleavage activity on one-site substrate in the absence of divalent metal ion and this activity was susceptible to external EDTA suggesting the presence of bound metal ion (Supplementary Figure S4). The last histidine residue in the HNH motif is known to act as a metal-binding residue. In case of HpyAII endonuclease histidine residue at 359 position (H359) aligned well with the metal binding residues of other HNH motif containing enzymes (Figure 1). In order to determine the role of H359 residue, the residue was replaced with alanine or glutamine. The activity of mutant proteins (H359A and H359Q) was checked on one-site oligonucleotide substrate in the presence and absence of divalent metal ions. It was observed that H359A mutant of HpyAII was unable to cleave one-site substrate in presence of Mg^2+^ ions. Moreover, no detectable cleavage was seen in the absence of Mg^2+^ ions with H359A mutant (Figure 6) whereas, H359Q HpyAII mutant was active and cleaved one-site substrate in the presence of Mg^2+^ ions. Similar level of cleavage was observed with wildtype HpyAII and H359Q mutant protein (Figure 6). The H359Q mutant also cleaved the DNA substrate in the absence of external metal ion. These results suggest that the glutamine residue can substitute the function of histidine as a metal-binding residue.

## Discussion

HpyAII and MboII R–M systems are isoschizomers and recognize the sequence GAAGA/TCTTC. MboII endonuclease is a type IIS endonuclease with PD(X)_n_ (E/D)XK active site [16]. Evidences show that MboII endonuclease cleaves two target sites DNA with higher efficiency compared with single-site substrate. Both plasmid and oligonucleotide substrates carrying two-site were cleaved more efficiently compared with one-site substrate [16]. In the present study, activity of HpyAII was compared on the identical plasmid substrates used for MboII cleavage assays. It was observed that HpyAII was unable to cleave both one and two sites plasmid substrate under steady state reaction condition ([E] < [S]). However, under single turnover reaction conditions ([E] > [S]) HpyAII cleaved two-site plasmid DNA substrate faster than the one-site substrate. Due to rapid rate of hydrolysis, the half time of the reaction was difficult to monitor with two-site plasmid DNA substrate. Further analysis with one and two sites oligonucleotide substrates revealed the similar preference for two-sites for maximal activity. It was observed that HpyAII performed ten times faster cleavage of two-site oligonucleotide compared with one-site oligonucleotide. This kind of substrate preference is a peculiar property of Type IIS endonucleases. Previous studies have shown that two-sites are needed for the transient dimerization of the monomeric FokI type IIS endonuclease. Since the recognition site is asymmetric two monomeric FokI subunits are required for the cleavage of double-stranded DNA [17,22]. The other type IIS enzymes such as BspMI and Eco57I also need two sites for efficient cleavage. The similarity of substrate preference of HpyAII and MboII could be attributed to the similar reaction mechanism as observed for FokI. The rate-limiting step is likely the dimerization of the two monomeric-DNA bound species to form a transient active site which results in the cleavage of the DNA [17]. This could explain the low reactivity of HpyAII on one-site substrate compared with two-site substrate.

It is very well-known that restriction endonucleases with classical PD-X-(D/E)XK motif cleave DNA in presence of Mg^2+^ and Mn^2+^, whereas Ca^2+^ only supports DNA binding. However, restriction enzymes belonging to ββα-Me family show relaxed preference for divalent metal ions. HNH motif containing enzymes which are subgroup of ββα-Me family are catalytically active in both alkaline earth metals and transition metal ions [21,23]. Colicin E9 which is HNH motif containing non-specific nuclease, cleaves dsDNA in presence of Mg^2+^ and Ca^2+^, whereas Ni^2+^ only supports ssDNA cleavage [24]. *Serratia* endonuclease show maximal activity with Mg^2+^, however mutants with preference to Mn^2+^, Co^2+^ and Zn^2+^ are also been reported [25]. KpnI, a type IIP endonuclease with HNH motif cleaves DNA specifically in the presence of Ca^2+^ ions, whereas Mg^2+^, Mn^2+^ and Co^2+^ promotes promiscuous DNA cleavage [23]. HpyAV, a type IIS endonuclease from *H. pylori* strain 26695 was more active with Mn^2+^, Ni^2+^, Co^2+^ than with Mg^2+^ ions [21]. In this study, it was observed that HpyAII showed similar preference for transition metal ions for dsDNA cleavage. Cofactor requirement of HpyAII shows that the enzyme is less stringent in metal ion requirement and shows higher cleavage activity with transition metals (Ni^2+^, Cd^2+^, Co^2+^) over alkaline earth metals (Mg^2+^, Ca^2+^). HpyAII is the second known example of restriction endonuclease which can cleave dsDNA in presence of Ni^2+^ ions after HpyAV. This is interesting as Ni^2+^ ions are important to the biology of *H. pylori* and various studies have shown that *H. pylori* maintains a pool of Ni^2+^ for successful colonization of the host [26].

It is known that the first histidine residue adjacent to the invariant histidine residue of the HNH motif is associated with metal-ion binding and is shown to act as general acid in the catalysis. Previous studies have determined the specific role of this residue in metal-ion coordination and substitution of this residue leads to enzyme inactivation [21,24,27]. Mutational and sequence analysis of HpyAII confirms that the protein has an active HNH site for the cleavage of DNA. However, mutation of first His residue (general acid) of the HNH motif to Ala does not abolish the enzymatic activity, but instead causes loss of fidelity and non-specific activity compared with wildtype HpyAII. The H328A mutant was able to cleave both canonical and non-canonical DNA substrate. It was also observed that H328A mutant like the wildtype HpyAII preferred two-site plasmid over one-site plasmid for efficient cleavage suggesting similar mode of action. The novelty of observation lays in the fact that mutation of the general acid His residue of the HNH motif in other known enzymes always abolished the enzymatic activity. Previously, a point mutation of the secondary metal binding residue of the KpnI endonuclease resulted in specific cleavage. The wildtype KpnI has a star-activity in presence of Mg^2+^ ions. The D163I mutant of KpnI does not exhibit promiscuous activity in the presence of Mg^2+^ ions [28]. The high-sequence specificity of type II restriction endonuclease set them apart from the other non-specific nucleases. It has been proposed that the high sequence specificity in case of type II endonucleases is an end result of genetic recombination and mutations [29,30]. However, sequence specificity via genetic recombination is a well-established phenomenon [31–33]. The only evidence which suggests the role of point mutation in sequence specificity is in the case of KpnI endonuclease [28]. In case of KpnI a single mutation changed the enzyme specificity from promiscuous to specific. In the present study, results with HpyAII endonuclease provide another line of evidence where a single mutation converts the enzyme from specific into non-specific. Current study using HpyAII provides another insight of the role of point mutation in the evolution of the enzyme sequence specificity. It is possible that in *H. pylori* strain 26695 high sequence specificity of HpyAII is naturally achieved by point mutations.

*H. pylori* is unique as the organism maintains a large pool of R–M systems as well as shows high natural competence [34]. We have previously shown that HpyAII endonuclease limits transformation of large DNA fragment in *H. pylori* [8]. Also we have shown that HpyAII endonuclease is a strain-specific endonuclease in Indian *H. pylori* clinical isolates as well in the global *H. pylori* isolates. In *H. pylori* strains 26695 and J99, out of the 46 predicted phase variable genes, nine belong to R–M systems. The most common mechanism of phase variation in R–M systems is through mutation of simple tandem repeats found to occur in methyltransferase or endonuclease genes. HpyAII endonuclease gene is a phase variable type IIS endonuclease having multiple stretches of adenine (A) residues in the gene sequence [14]. This selective ON and OFF of endonuclease may play a significant role in the evolution of *H. pylori* by preventing or facilitating DNA uptake. This kind of adaptation can further enable *H. pylori* to make functional changes, which in turn give a selective advantage, to changes that occur perpetually during colonization and persistent infection.

## Supplementary Material

Supplementary Figures S1-S5

## Abbreviations

CD: circular dichroism
FLL: full-length linear fragment
IPTG: isopropyl-1-thio-β-d-galactopyranoside
LB: lysogeny broth
PCR: polymerase chain reaction
R–M: restriction–modification
SDM: site-directed mutagenesis

## References

[1] Krebes J., Morgan R.D., Bunk B., Sproer C., Luong K., Parusel R. et al. (2014) The complex methylome of the human gastric pathogen *Helicobacter pylori*. Nucleic Acids Res. 42, 2415–2432 10.1093/nar/gkt1201 24302578 PMC3936762

[2] Furuta Y., Namba-Fukuyo H., Shibata T.F., Nishiyama T., Shigenobu S., Suzuki Y. et al. (2014) Methylome diversification through changes in DNA methyltransferase sequence specificity. PLoS Genet. 10, e1004272 10.1371/journal.pgen.1004272 24722038 PMC3983042

[3] Heusipp G., Falker S. and Schmidt M.A. (2007) DNA adenine methylation and bacterial pathogenesis. Int. J. Med. Microbiol. 297, 1–7 10.1016/j.ijmm.2006.10.002 17126598

[4] Kumar R., Mukhopadhyay A.K., Ghosh P. and Rao D.N. (2012) Comparative transcriptomics of *H. pylori* strains AM5, SS1 and their hpyAVIBM deletion mutants: possible roles of cytosine methylation. PLoS ONE 7, e42303 10.1371/journal.pone.0042303 22879937 PMC3411764

[5] Skoglund A., Bjorkholm B., Nilsson C., Andersson A.F., Jernberg C., Schirwitz K. et al. (2007) Functional analysis of the M.HpyAIV DNA methyltransferase of *Helicobacter pylori*. J. Bacteriol. 189, 8914–8921 10.1128/JB.00108-07 17921292 PMC2168601

[6] Srikhanta Y.N., Gorrell R.J., Steen J.A., Gawthorne J.A., Kwok T., Grimmond S.M. et al. (2011) Phasevarion mediated epigenetic gene regulation in *Helicobacter pylori*. PLoS ONE 6, e27569 10.1371/journal.pone.0027569 22162751 PMC3230613

[7] Srikhanta Y.N., Fox K.L. and Jennings M.P. (2010) The phasevarion: phase variation of type III DNA methyltransferases controls coordinated switching in multiple genes. Nat. Rev. Microbiol. 8, 196–206 10.1038/nrmicro2283 20140025

[8] Kumar S., Karmakar B.C., Nagarajan D., Mukhopadhyay A.K., Morgan R.D. and Rao D.N. (2018) N4-cytosine DNA methylation regulates transcription and pathogenesis in *Helicobacter pylori*. Nucleic Acids Res. 46, 3815 10.1093/nar/gky195 29538771 PMC5909436

[9] Estibariz I., Overmann A., Ailloud F., Krebes J., Josenhans C. and Suerbaum S. (2019) The core genome m5C methyltransferase JHP1050 (M.Hpy99III) plays an important role in orchestrating gene expression in *Helicobacter pylori*. Nucleic Acids Res. 47, 2336–2348 10.1093/nar/gky1307 30624738 PMC6412003

[10] Nobusato A., Uchiyama I. and Kobayashi I. (2000) Diversity of restriction-modification gene homologues in *Helicobacter pylori*. Gene 259, 89–98 10.1016/S0378-1119(00)00455-8 11163966

[11] Kong H., Lin L.F., Porter N., Stickel S., Byrd D., Posfai J. et al. (2000) Functional analysis of putative restriction-modification system genes in the *Helicobacter pylori* J99 genome. Nucleic Acids Res. 28, 3216–3223 10.1093/nar/28.17.3216 10954588 PMC110709

[12] Nobusato A., Uchiyama I., Ohashi S. and Kobayashi I. (2000) Insertion with long target duplication: a mechanism for gene mobility suggested from comparison of two related bacterial genomes. Gene 259, 99–108 10.1016/S0378-1119(00)00456-X 11163967

[13] Aras R.A., Takata T., Ando T., van der Ende A. and Blaser M.J. (2001) Regulation of the HpyII restriction-modification system of *Helicobacter pylori* by gene deletion and horizontal reconstitution. Mol. Microbiol. 42, 369–382 10.1046/j.1365-2958.2001.02637.x 11703661

[14] Lin L.F., Posfai J., Roberts R.J. and Kong H. (2001) Comparative genomics of the restriction-modification systems in *Helicobacter pylori*. Proc. Natl. Acad. Sci. U.S.A. 98, 2740–2745 10.1073/pnas.051612298 11226310 PMC30209

[15] Lippow S.M., Aha P.M., Parker M.H., Blake W.J., Baynes B.M. and Lipovsek D. (2009) Creation of a type IIS restriction endonuclease with a long recognition sequence. Nucleic Acids Res. 37, 3061–3073 10.1093/nar/gkp182 19304757 PMC2685105

[16] Soundararajan M., Chang Z., Morgan R.D., Heslop P. and Connolly B.A. (2002) DNA binding and recognition by the IIs restriction endonuclease MboII. J. Biol. Chem. 277, 887–895 10.1074/jbc.M109100200 11606594

[17] Pernstich C. and Halford S.E. (2012) Illuminating the reaction pathway of the FokI restriction endonuclease by fluorescence resonance energy transfer. Nucleic Acids Res. 40, 1203–1213 10.1093/nar/gkr809 21993298 PMC3273807

[18] Shevchenko A., Tomas H., Havlis J., Olsen J.V. and Mann M. (2006) In-gel digestion for mass spectrometric characterization of proteins and proteomes. Nat. Protoc. 1, 2856–2860 10.1038/nprot.2006.468 17406544

[19] Tabor S. (2001) Expression using the T7 RNA polymerase/promoter system. Curr. Protoc. Mol. Biol. Chapter 16, Unit16.2 10.1002/0471142727.mb1602s11 18265127

[20] Bath A.J., Milsom S.E., Gormley N.A. and Halford S.E. (2002) Many type IIs restriction endonucleases interact with two recognition sites before cleaving DNA. J. Biol. Chem. 277, 4024–4033 10.1074/jbc.M108441200 11729187

[21] Chan S.H., Opitz L., Higgins L., O’Loane D. and Xu S.Y. (2010) Cofactor requirement of HpyAV restriction endonuclease. PLoS ONE 5, e9071 10.1371/journal.pone.0009071 20140205 PMC2816704

[22] Bitinaite J., Wah D.A., Aggarwal A.K. and Schildkraut I. (1998) FokI dimerization is required for DNA cleavage. Proc. Natl. Acad. Sci. U.S.A. 95, 10570–10575 10.1073/pnas.95.18.10570 9724744 PMC27935

[23] Vasu K., Saravanan M. and Nagaraja V. (2011) Endonuclease active site plasticity allows DNA cleavage with diverse alkaline Earth and transition metal ions. ACS Chem. Biol. 6, 934–942 10.1021/cb200107y 21736285

[24] Pommer A.J., Cal S., Keeble A.H., Walker D., Evans S.J., Kuhlmann U.C. et al. (2001) Mechanism and cleavage specificity of the H-N-H endonuclease colicin E9. J. Mol. Biol. 314, 735–749 10.1006/jmbi.2001.5189 11733993

[25] Friedhoff P., Kolmes B., Gimadutdinow O., Wende W., Krause K.L. and Pingoud A. (1996) Analysis of the mechanism of the Serratia nuclease using site-directed mutagenesis. Nucleic Acids Res. 24, 2632–2639 10.1093/nar/24.14.2632 8758988 PMC146012

[26] Benoit S.L., Miller E.F. and Maier R.J. (2013) *Helicobacter pylori* stores nickel to aid its host colonization. Infect. Immun. 81, 580–584 10.1128/IAI.00858-12 23230291 PMC3553817

[27] Walker D.C., Georgiou T., Pommer A.J., Walker D., Moore G.R., Kleanthous C. et al. (2002) Mutagenic scan of the H-N-H motif of colicin E9: implications for the mechanistic enzymology of colicins, homing enzymes and apoptotic endonucleases. Nucleic Acids Res. 30, 3225–3234 10.1093/nar/gkf420 12136104 PMC135741

[28] Saravanan M., Vasu K. and Nagaraja V. (2008) Evolution of sequence specificity in a restriction endonuclease by a point mutation. Proc. Natl. Acad. Sci. U.S.A. 105, 10344–10347 10.1073/pnas.0804974105 18647833 PMC2492507

[29] Arber W. and Linn S. (1969) DNA modification and restriction. Annu. Rev. Biochem. 38, 467–500 10.1146/annurev.bi.38.070169.002343 4897066

[30] Arber W. (2000) Genetic variation: molecular mechanisms and impact on microbial evolution. FEMS Microbiol. Rev. 24, 1–7 10.1111/j.1574-6976.2000.tb00529.x 10640595

[31] Fuller-Pace F.V., Bullas L.R., Delius H. and Murray N.E. (1984) Genetic recombination can generate altered restriction specificity. Proc. Natl. Acad. Sci. U.S.A. 81, 6095–6099 10.1073/pnas.81.19.6095 6091134 PMC391866

[32] Fuller-Pace F.V. and Murray N.E. (1986) Two DNA recognition domains of the specificity polypeptides of a family of type I restriction enzymes. Proc. Natl. Acad. Sci. U.S.A. 83, 9368–9372 10.1073/pnas.83.24.9368 3025838 PMC387139

[33] Nagaraja V., Shepherd J.C. and Bickle T.A. (1985) A hybrid recognition sequence in a recombinant restriction enzyme and the evolution of DNA sequence specificity. Nature 316, 371–372 10.1038/316371a0 2991768

[34] Dorer M.S., Cohen I.E., Sessler T.H., Fero J. and Salama N.R. (2013) Natural competence promotes *Helicobacter pylori* chronic infection. Infect. Immun. 81, 209–215 10.1128/IAI.01042-12 23115044 PMC3536137

