# Supplementary material for: Promiscuous DNA cleavage by HpyAII endonuclease is modulated by the HNH catalytic residues
Source: Biosci Rep. 2020 Sep 16;40(9):BSR20201633. doi: 10.1042/BSR20201633 (PMC7494987; doi:10.1042/BSR20201633)
Supplement: Supplementary Figures S1-S5 [file BSR-2020-1633_supp.pdf]

Figure S1

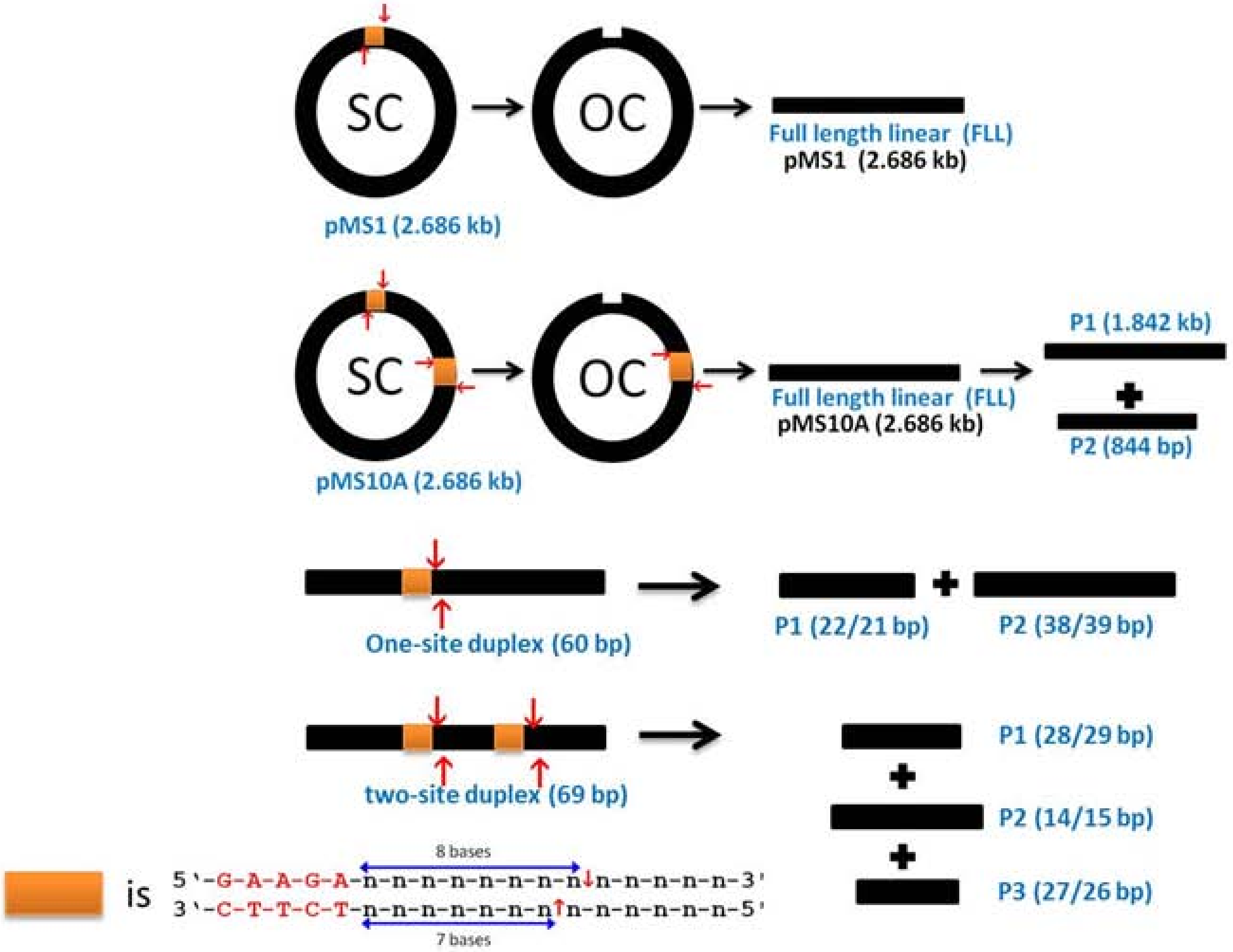

DNA substrates used in the present study. Cleavage of one-site plasmid (pMS1) will generate a final linear product of 2.686 kb with open-circular plasmid as an intermediate. Two-site plasmid (pMS10A) upon complete cleavage will generate two linear fragments of size 1.842 kb and 844 bp with two intermediates cleavage (open-circular and full length linear) products. Cleavage of one-site oligonucleotide duplex (60 bp) will results in two fragments. Complete cleavage of two-site oligonucleotide duplex will produce three fragments.

Figure S2

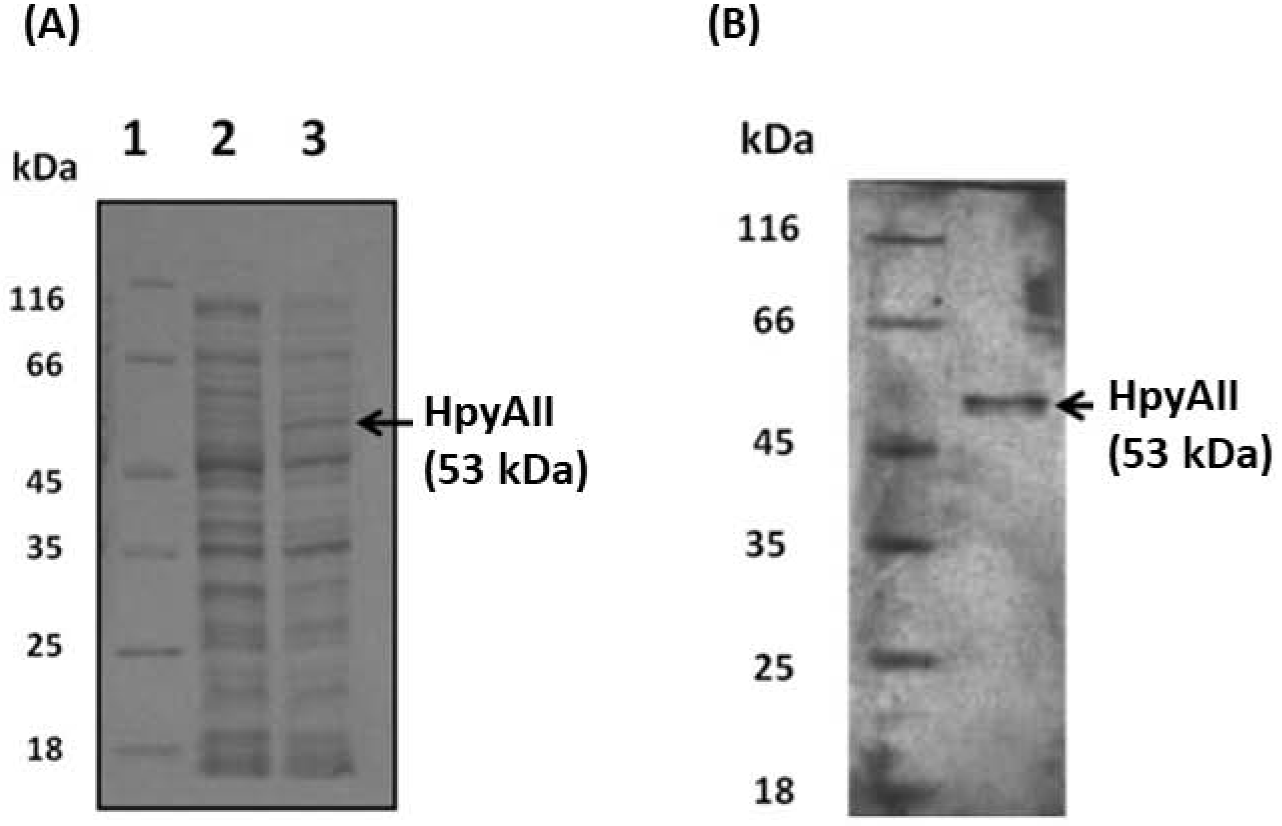

Overexpression and purification of N-terminal His$_6$-tag HpyAII endonuclease (~53 kDa). (A) Overexpression of 53 kDa HpyAII was done in *E.coli Rosetta* (DE3) pLysS cells with 1 mM IPTG. Lane 1: Protein molecular weight ladder, lane 2: cell lysate from uninduced cell lysate of *E.coli* Rosetta (DE3) pLysS cells transformed with pET28a-*R.hpyAII* vector, lane 3: induced sample after 2 hours induction with 1 mM IPTG. **(B)** Purification of HpyAII endonuclease using Ni^{2+}-NTA resin followed by Heparin-sepharose column purification. Lane 1: protein molecular weight ladder, lane 2: purified HpyAII endonuclease. Protein purity was checked by performing silver staining using 5 μg purified HpyAII endonuclease.

Figure S3

(A)

(B)

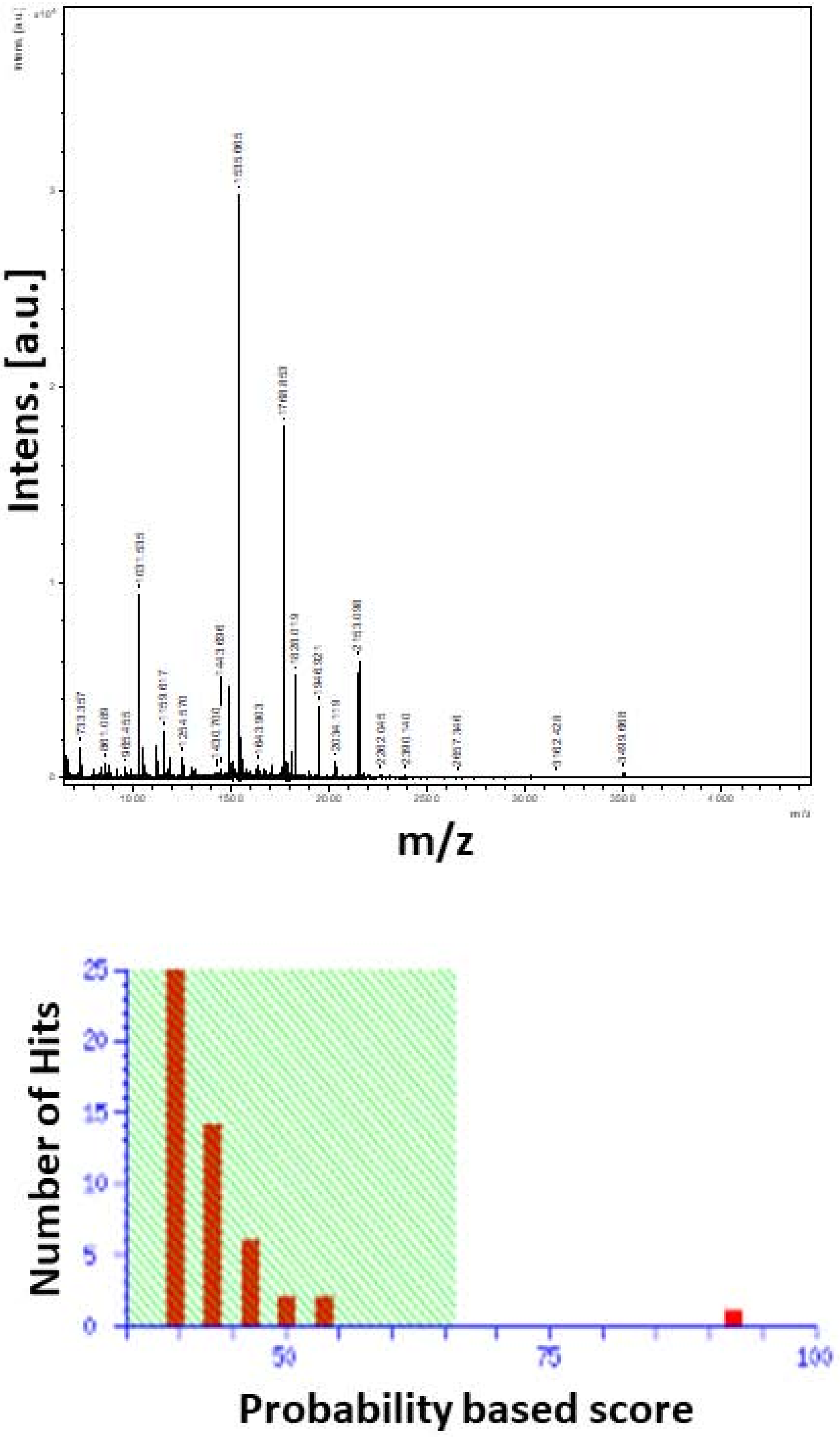

Peptide mass fingerprinting of purified HpyAII endonuclease.
(A) Peptide mass fingerprinting spectra of purified HpyAII endonuclease (53 kDA). Peptide mass fingerprinting was done using trypsin protease. **(B)** Mascot scores ($P < 0.05$, number of matching peptides= 38). Database search using the mass list of the experiment produced a significant match for HpyAII endonuclease from *H. pylori* strain 26695.

Figure S4

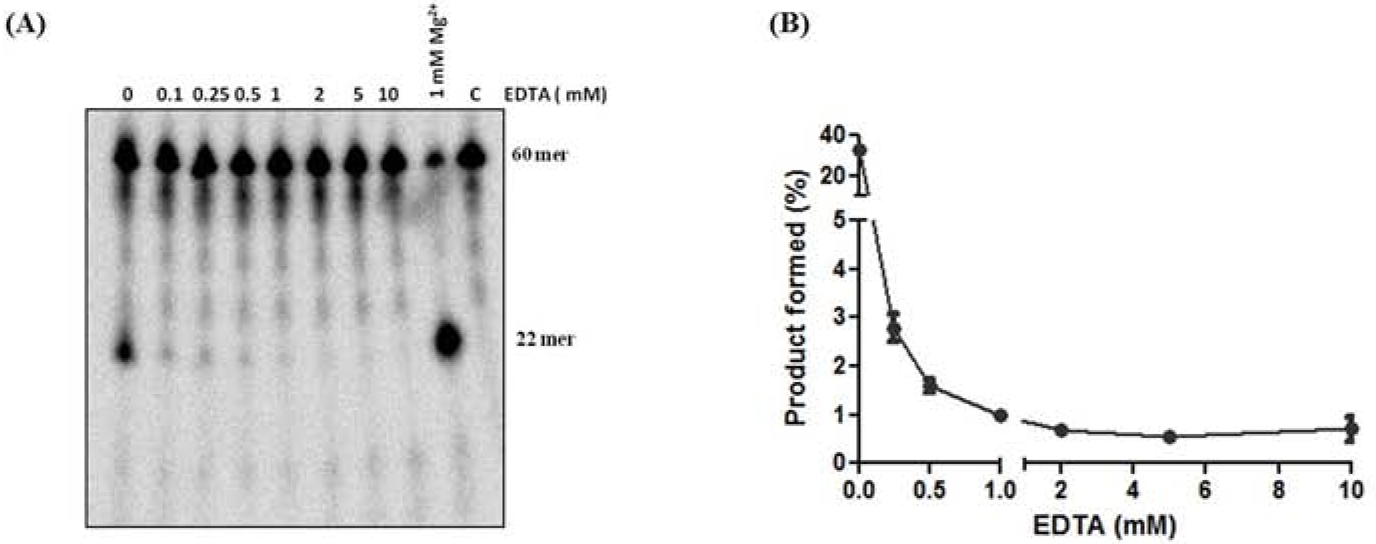

Effect of EDTA on the endonuclease activity of HpyAII endonuclease. (A) Endonuclease activity of HpyAII endonuclease on radiolabeled 60 bp DNA was carried out at various concentrations of EDTA (0.1-10 mM) in the cleavage buffer lacking divalent metal ions. 1 mM Mg2+ was used as an internal control for cleavage assay. Cleavage assay was perfomed for 30 minutes at 37°C and assay was stopped by the addition of reaction stop mixture containing 20 mM EDTA. **(B)** Quantitative analysis of product formed (%) with respect to EDTA concentration (mM).

Figure S5

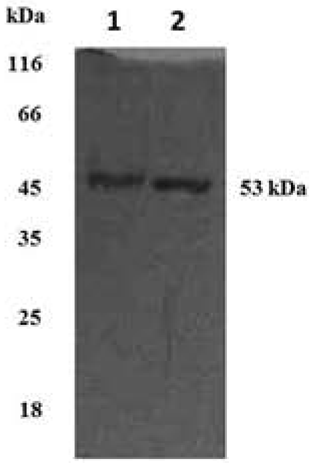

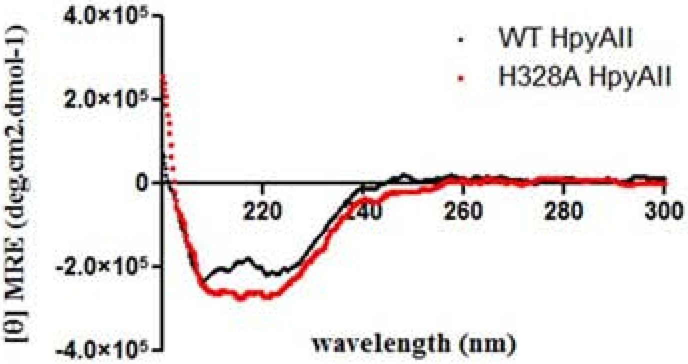

Purification of H328A HpyAII and Circular dichroism (CD) spectra of HpyAII and H328A HpyAII proteins. (A) HpyAII and H328A HpyAII was purified as described in Materials and methods section. SDS-PAGE analysis of purified HpyAII and H328A mutant. Lane 1: HpyAII, Lane 2: H328A HpyAII. **(B)** CD spectra of HpyAII and H328A proteins. HpyAII and H328A (0.1mg/ mL) were dialysed in 10 mM Tris-Cl buffer, pH 7.4. The CD spectra were recorded at scan speed of 100 nm per min in the wavelength range 190-300 nm. Experiment was done at 25°C.
